# Embryonic Cells Redistribute SUMO1 upon Forced SUMO1 Overexpression

**DOI:** 10.1128/mBio.01856-19

**Published:** 2019-12-03

**Authors:** Andreia Lee, Yiping Zhu, Yosef Sabo, Stephen P. Goff

**Affiliations:** a Department of Biological Sciences, Howard Hughes Medical Institute, Columbia University Medical Center, Columbia University, New York, New York, USA; b Department of Biochemistry and Molecular Biophysics, Howard Hughes Medical Institute, Columbia University Medical Center, Columbia University, New York, New York, USA; c Department of Microbiology and Immunology, Howard Hughes Medical Institute, Columbia University Medical Center, Columbia University, New York, New York, USA; d Department of Medicine, Howard Hughes Medical Institute, Columbia University Medical Center, Columbia University, New York, New York, USA; University of Washington

**Keywords:** SUMO, embryonic stem cells, sumoylation, posttranslational modification

## Abstract

Conjugation of small ubiquitin-like modifiers (SUMOs) to substrate proteins is a posttranslational protein modification that affects a diverse range of physiological processes. Global inhibition of SUMO conjugation in mice results in embryonic lethality, reflecting the importance of the SUMO pathways for embryonic development. Here, we demonstrated that SUMO1 overexpression was not well tolerated in murine embryonic carcinoma and embryonic stem (ES) cells and that only a few clones were recovered after transduction with vectors delivering SUMO1 expression constructs. Differentiated NIH/3T3 cells overexpress SUMO1 without deleterious effects and maintain high levels of both conjugated and free forms of SUMO1. The few embryonic cells surviving after forced overexpression retained all their SUMO1 in the form of a few high-molecular-weight conjugates and maintained undetectable levels of free SUMO1. The absence of free SUMO in embryonic cells was seen specifically upon overexpression of SUMO1, but not SUMO2. Moreover, blocking SUMO1 conjugation to endogenous substrates by C-terminal mutations of SUMO1 or by overexpression of a SUMO1 substrate “sponge” or by overexpression of the deSUMOylating enzyme SUMO-specific peptidase 1 (SENP1) dramatically restored free SUMO1 overexpression. The data suggest that overexpression of SUMO1 protein leading to an excess accumulation of critical SUMO1-conjugated substrates is not tolerated in embryonic cells. Surviving embryonic cells exhibit SUMO1 conjugation to allowed substrates but a complete absence of free SUMO1.

## Abstract (precis)

Embryonic stem (ES) cells exhibit unusual transcriptional, proteomic, and signal response profiles, reflecting their unusual needs for rapid differentiation and replication. The work reported here demonstrated that mouse embryonic cell lines did not tolerate the overexpression of SUMO1, the small ubiquitin-like modifier protein that is covalently attached to many substrates to alter their intracellular localization and functionality. Forced SUMO1 overexpression is toxic to ES cells, and surviving cell populations adapt by dramatically reducing the levels of free SUMO1. Such a response is not seen in differentiated cells or with SUMO2 or with nonconjugatable SUMO1 mutants or in the presence of a SUMO1 “sponge” substrate that accepts the modification. The findings suggest that excess SUMO1 modification of specific substrates is not tolerated by embryonic cells and highlight a distinctive need for these cells to control the levels of SUMO1 available for conjugation.

## INTRODUCTION

The conjugation of small ubiquitin-like modifiers (SUMOs) to protein substrates is a posttranslational modification that impacts a diverse range of cellular processes such as transcriptional regulation, RNA processing, viral repression, the DNA damage response, and protein localization (1–3). The downstream consequences of SUMO conjugation are quite varied and are substrate specific. Sumoylation or desumoylation can result in alterations in protein-protein interactions, protein-DNA interactions, protein stability, protein trafficking, or protein activity (3). Several important mammalian proteins are modified and regulated by sumoylation, including Ran GTPase-activating protein 1 (RanGAP1), p53, c-Jun, tripartite motif-containing 28 (Trim28 or KAP1), and histone deacetylase 1 (HDAC1) (4–6), and the list of proteins modified by SUMO continues to expand. Recent proteomic studies reported estimates that up to 15% of human proteins may be modified by SUMO (7). Aberrations in the SUMO pathway can result in tumor development and progression, heart defects, and Alzheimer’s disease (8–10). Thus, a growing body of work is highlighting the importance of SUMO for normal cellular function.

The SUMO family in mammals consists of four members: SUMO1, SUMO2, SUMO3, and SUMO4. SUMO1 is widely expressed in many cell types and is by far the best-characterized family member. SUMO2 and SUMO3 share only 50% sequence identity with SUMO1 and often perform distinct cellular functions. They are 97% identical to each other and often modify the same substrates, though they clearly have different roles in development (11). SUMO4 is expressed only in the kidney, dendritic cells, and macrophages (1, 12). Some substrates can be conjugated by any of the SUMOs, while others accept only specific SUMOs (13). The significance of this selectivity is unclear. The SUMOs are often conjugated to their substrates as monomers, but polymeric chains can also be formed (3).

Targets of sumoylation commonly contain the tetrapeptide consensus motif Ψ-K-x-D/E, where Ψ is a hydrophobic residue, K is the lysine directly conjugated by SUMO, x is any amino acid, and D/E is an acidic residue. However, 50% of SUMO conjugation occurs on lysine residues that do not adhere to this consensus sequence (14). Prior to conjugation, the SUMOs must be activated by cleavage at the C terminus by SUMO-specific peptidase (SENP) proteases, exposing diglycine residues needed for transfer. Mature SUMOs are covalently conjugated to lysine residues of substrates in a cascade mediated by three enzymes—an activating enzyme, SAE1/2 (E1); a conjugating enzyme, Ubc9 (E2); and one of many targeting ligases (E3). SENPs also function in the deconjugation of the SUMOs and are responsible for rapid cycles of conjugation and deconjugation of SUMOs from its substrates. Six SUMO-specific peptidase (SENP) proteases (SENP1, SENP2, SENP3, SENP5, SENP6, and SENP7) are found in mice and human. SENP1 can deconjugate SUMO1 as well as SUMO2 and SUMO3, while SENP2, SENP3, SENP5, SENP6, and SENP7 are predominantly responsible for deconjugation of SUMO2 and SUMO3 (15, 16). Desumoylation by the SENP family is a key process for regulating the steady-state levels of a SUMO-modified substrate, which generally makes up less than 5% of the total substrate protein (3).

SUMO modifications of many proteins, including specific transcription factors, are important in embryonic development (17). The SUMO E2 ligase Ubc9 is essential for induction of induced pluripotent stem (iPS) cells and for survival of ES cells (18). Knockout (KO) of Ubc9, likely eliminating all SUMO conjugation, results in death of early embryos at the postimplantation stage (19). Surprisingly, however, SUMO1 KO mice develop normally, indicating that SUMO1 modification *per se* is not essential (11, 13). In contrast, SUMO2 KO mice are not viable, indicating that SUMO2 is an essential family member (11). While loss of SUMO1 is tolerated, excessive conjugation of SUMO1 is apparently toxic. KO of the deconjugating enzyme SENP1 is embryonically lethal, and this lethality can be rescued by genetically reducing SUMO1 levels (16). The identity of the overSUMOylated substrate(s) that causes the lethality is not known.

Here, we investigated the effects of SUMO overexpression in mammalian cell lines and showed that embryonic cells, but not differentiated cells, cannot readily tolerate overexpression of SUMO1 protein capable of conjugation to substrates. Surviving cells have redistributed their SUMO1 and no longer maintain free SUMO1. In contrast, SUMO2 was readily overexpressed in both embryonic and differentiated cells. Reducing SUMO1 conjugation by eliminating the diglycine residues necessary for conjugation or by coexpression of a “SUMO sponge” or by coexpression of the desumoylase SENP1 greatly improved overexpression of free SUMO1. The results suggest that embryonic cells do not tolerate the excessive formation of the critical SUMO1-conjugated substrate(s).

## RESULTS

### SUMO1 cannot be overexpressed to accumulate as free SUMO1 in embryonic cells.

**FIG 1:**
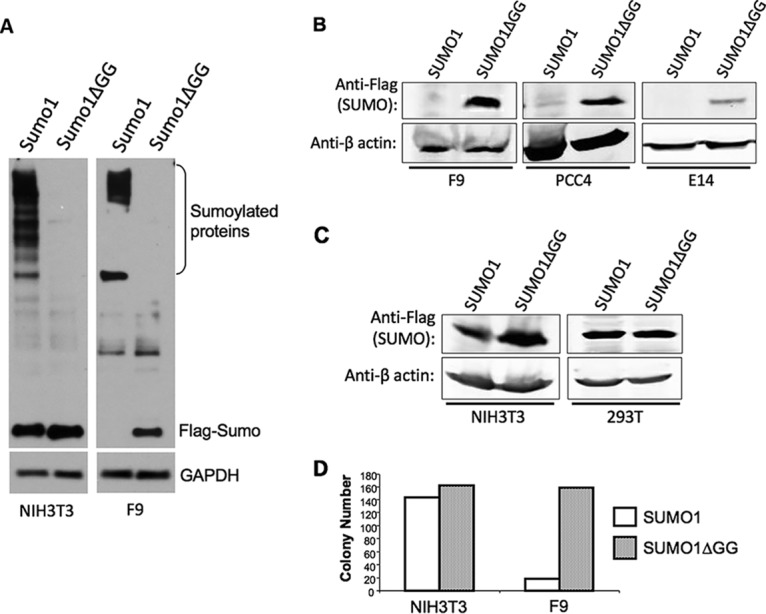
Accumulation of free SUMO1 is specifically blocked in embryonic cell lines. (A) Western blot of NIH 3T3 or F9 cells after transduction with viral vectors delivering wild-type Flag-SUMO1 or mutant Flag-SUMO1 lacking the six C-terminal residues (SUMO1ΔGG). Cell lysates were prepared using relatively harsh RIPA buffer. The positions of free SUMO1 and high-molecular-weight conjugates are indicated. The blot was reprobed for GAPDH (glyceraldehyde-3-phosphate dehydrogenase) as loading control. (B) Western blot of lysates of embryonic cell lines (F9, PCC4, and E14 cells) transduced with vectors expressing either Flag-SUMO1 or Flag-SUMO1ΔGG as indicated, selected for puromycin resistance encoded by the vector. The blot was probed with anti-Flag antibodies or anti-actin antibodies as a loading control, as indicated. (C) Western blot of differentiated cell lines (NIH 3T3 and 293T cells) transduced with vectors expressing Flag-SUMO1 or Flag-SUMO1ΔGG. The blot was probed with anti-Flag antibodies or anti-actin antibodies as a loading control, as indicated. (D) Efficiency of colony formation after transduction of NIH 3T3 or F9 cells by vectors expressing either wild-type SUMO1 or mutant SUMO1ΔGG. Cells were exposed to equal concentrations of virus preparations and were plated in medium with puromycin. Colonies per 10-cm-diameter dish were counted after 10 days.

Many studies have suggested that SUMOylation has a uniquely significant role in embryonic development (17, 18) and thus might be subject to distinctive regulation in developmentally primitive cell types. To examine the consequences of increased SUMOylation in embryonic cells, we designed DNA constructs that would drive high-level expression of SUMO1. Because embryonic cells are difficult to transfect and can silence a variety of promoters, we delivered the constructs on lentiviral vector genomes in which the EF1α promoter, active in embryonic cells, drove expression of Flag-tagged SUMO1 and a drug resistance protein (PuroR) designed to be translated from a single bicistronic transcript. The SUMO1 gene was positioned at the 5′ end of the transcript so as to be translated by cap-dependent ribosome initiation events, while the 3′ proximal puromycin resistance gene was translated separately by ribosomes initiating at an internal ribosome entry site (IRES). Constructs were generated encoding Flag-tagged versions of either a wild-type (WT) full-length SUMO1 precursor, requiring processing for conjugation (Flag-SUMO1), or a truncated version lacking the six C-terminal residues, including the GG residues needed for ligation (Flag-SUMO1ΔGG). 293T cells were transfected with these vector DNAs, along with pCMVΔR8.2 DNA encoding the HIV-1 Gag and Gag-Pol proteins and pVSV-G DNA expressing the vesicular stomatitis virus G (VSV-G) envelope protein, and viral particles in the culture supernatants were collected. The virus preparations were applied to NIH 3T3 cells or F9 embryonic carcinoma cells, and transduced cells were selected with puromycin. Lysates of the pooled transduced cell cultures were prepared using harsh buffer conditions, and the levels of expression of SUMO1 were then assessed by Western blotting probed with anti-Flag antibodies. NIH 3T3 cells transduced with the wild-type SUMO1 vector accumulated both a spectrum of high-molecular-weight SUMO1 conjugates and free monomeric SUMO1 (Fig. 1A). In contrast, F9 cells transduced with the wild-type SUMO1 expressed no detectable free SUMO1 but retained all the SUMO1 in form of a few high-molecular-weight species (Fig. 1A). Many of the bands seen in NIH 3T3 cells were absent in the F9 cells. Both cell lines transduced with the SUMO1ΔGG construct, however, expressed high levels of the free monomeric mutant SUMO1.

The complete absence of free wild-type SUMO1 accumulation was seen with other embryonic cells. Transduction of the PCC4 embryonic carcinoma line or the E14 embryonic stem cell line gave results similar to those seen in F9 cells: no free wild-type SUMO1 but high levels of free SUMO1ΔGG (Fig. 1B). We note that the E14 line expressed the transduced SUMO1ΔGG at lower levels than the other cell lines, though again at much higher levels than the wild-type SUMO1. Delivering the same constructs into differentiated cell lines, both the mouse NIH 3T3 fibroblast line and human 293T cells resulted in high and comparable levels of expression of the free monomeric forms of both SUMO1 and SUMO1ΔGG (Fig. 1C).

One possible explanation for the low expression of free wild-type SUMO1 in embryonic cells is that high expression is toxic and that only very few transduced cells with aberrant SUMO1 processing were surviving the drug selection. To test this, the efficiency of recovery of F9 cells after transduction was examined. Equal concentrations of virus preparations expressing wild-type SUMO1 and SUMO1ΔGG were applied to NIH 3T3 or F9 cells, the cells were plated in medium with puromycin, and the numbers of drug-resistant colonies were determined. NIH 3T3 cells yielded comparable numbers of colonies after transduction by wild-type SUMO1 or SUMO1ΔGG viruses. F9 cells, in contrast, yielded approximately 8-fold-fewer colonies after infection with the wild-type SUMO1 virus than after infection with the mutant SUMO1ΔGG virus (Fig. 1D). The resulting transduced cells were pooled and passaged for long-term culture, and the morphologies and rates of growth of the resulting drug-resistant F9 cell populations were not distinguishable. The results suggest that embryonic cells were distinctly sensitive to overexpression of SUMO1, with few clones surviving to form colonies. The few surviving clones had blocked the accumulation of unconjugated SUMO1 protein while retaining all the SUMO1 in a few high-molecular-weight conjugates. In contrast, SUMO1ΔGG was expressed in F9 cells without the equivalent toxic effects on cell survival and was fully retained in free unconjugated form.

### Accumulation of free SUMO1 in embryonic cells is prevented at the posttranscriptional level.

**FIG 2:**
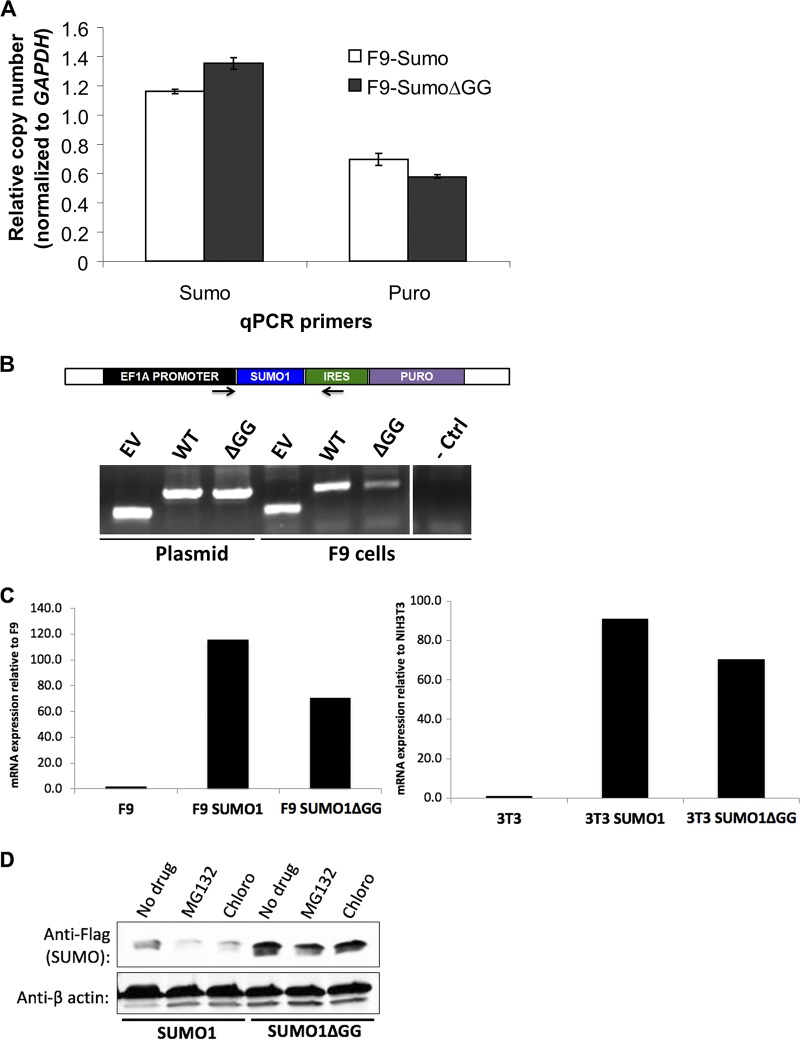
SUMO1 DNAs are retained and SUMO1 mRNAs are expressed at normal levels after transduction with vectors expressing SUMO1 or SUM1ΔGG. (A) qPCR of SUMO1 and puromycin resistance transgene levels in DNA isolated from F9 cells transduced with vectors expressing SUMO1 or SUMO1ΔGG as indicated. Copy numbers are expressed relative to GAPDH copy number, with both calculated from the PCR cycles at half-maximum signal. (B) Schematic of primers locations used for qPCR for SUMO1 sequences on transducing vector. Gel electrophoresis of PCR amplification products of SUMO insertions from plasmid DNA and DNA from F9 cells transduced with empty vector (EV), SUMO1, or SUMO1ΔGG as indicated. - Ctrl, negative control. (C) qRT-PCR of exogenous SUMO1 and SUMO1ΔGG mRNA levels in F9 cells (left) and NIH 3T3 cells (right). Expression levels are shown relative to levels in untransduced cells. (D) F9 cells were transduced with vectors expressing SUMO1 or SUM1ΔGG as indicated and were then either left untreated (No drug) or treated with proteasome inhibitor MG132 or lysosomal protease inhibitor chloroquine (Chloro) as indicated.

To probe the basis for the restriction in accumulation of free SUMO1, we examined the levels of SUMO1 DNA and RNA in transduced cell populations. F9 or 293T cells were transduced with the SUMO1 or SUMO1ΔGG vectors, or empty vector control, and were selected with puromycin for stable expression of the drug selection marker. The levels of SUMO1 and SUMO1ΔGG transgene DNAs were assessed by quantitative PCR (qPCR) using primers spanning exon-exon junctions, such that DNA of the endogenous SUMO1 gene would not be amplified (Fig. 2A). Similar levels of the SUMO1 and SUMO1GG transgenes per cell were found in the F9 and 293T pooled drug-resistant cells. SUMO1 transgene DNA was not detected in control F9 cells transduced by the empty vector. We also measured the levels of the drug resistance marker DNA (puromycin resistance gene), and as expected, levels of the puromycin resistance gene were similar across all transduced cells. The levels of the SUMO1 and *puroR* DNAs normalized for the differential efficiencies of amplification seen with qPCR of the original SUMO1 vector DNA and assessed by qPCR were comparable. Thus, the SUMO1 expression construct was correctly delivered to the embryonic cells and was retained in the surviving drug-resistant clones.

To test for the possibility that mutations in the SUMO1 vector were being selected during transduction, we examined the amplified PCR products from the pools of transduced cells. Genomic DNA was isolated from F9 cells transduced with the SUMO1 or SUMO1ΔGG vectors, and the SUMO1 insertion was amplified by PCR using primers that spanned the EF1a promoter and QQ region. PCR products from F9 cells transduced with SUMO1, SUMO1ΔGG, or empty vector were identical in size to the PCR products amplified from the corresponding plasmid controls (Fig. 2B). The bulk PCR products amplified from F9 cells were purified and sequenced, and no mutations differing from the wild-type sequence were detected in the SUMO1 transgenes in the pooled DNAs.

To examine the SUMO1 and SUMO1ΔGG transcript levels, we isolated RNA from F9 and NIH 3T3 cells transduced with the SUMO1 or SUMO1ΔGG vectors. RNAs were also isolated from untreated F9 and NIH 3T3 cells as negative controls. cDNA was synthesized from the RNAs, and SUMO1 or SUMO1ΔGG RNA levels were assessed by reverse transcription-quantitative PCR (qRT-PCR) using primers spanning SUMO1 exon-exon junctions. We detected similarly high levels of SUMO1 and SUMO1ΔGG transcripts in transduced F9 cells and NIH 3T3 cells and only very low levels in the untransduced control cells (Fig. 2C). Thus, the differences in the levels of utilization of the SUMO1 produced from these two constructs in embryonic cells were not due to unequal levels of transcripts.

One possible explanation for the lack of free SUMO1 accumulation in embryonic cells is protein degradation via either the proteasomal or lysosomal pathway. To test whether SUMO1 is degraded in embryonic cells by either pathway, F9 cells were transduced with the SUMO1 or SUMO1ΔGG vectors, and at 72 h postinfection the cells were treated with the proteasome inhibitor MG132 or the lysosomal degradation inhibitor chloroquine for differing times. Cell lysates were prepared and analyzed for SUMO1 expression by Western blotting. Free SUMO1 levels did not increase after 4 h of treatment with either drug (Fig. 2D). Prolonging drug treatment to 8 h, 12 h, or 24 h did not increase the low levels of SUMO1 expression (data not shown). The results suggest that proteasomal or lysosomal degradation was probably not responsible for the low levels of SUMO1 expression.

### SUMO2 can be overexpressed in embryonic and differentiated cells.

**FIG 3:**
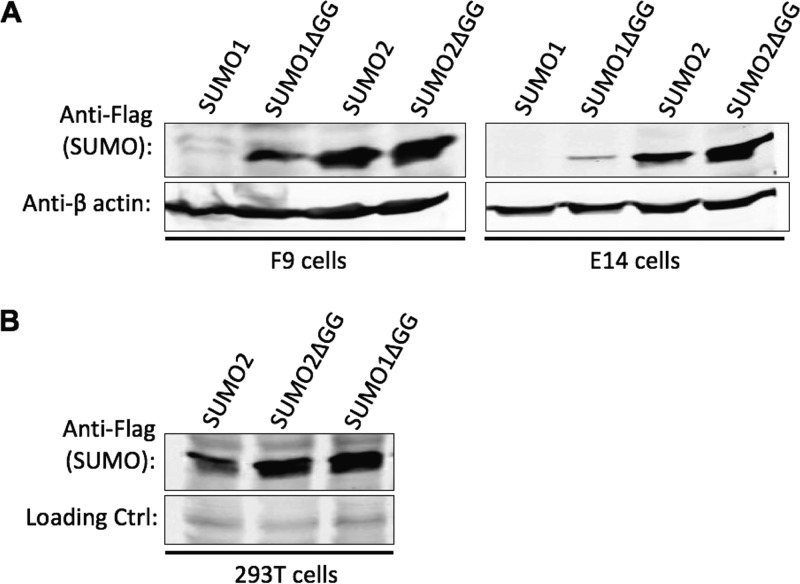
SUMO2 is overexpressed without change in status from the free form to the conjugated form in embryonic cells. (A) Western blotting of embryonic F9 and E14 cells as indicated, transduced with vectors expressing Flag-SUMO2 or Flag-SUMO2ΔGG. Flag-SUMO1 and Flag-SUMO1ΔGG cells are included for comparison. Blots were probed with anti-Flag or anti-actin antibodies as a loading control as indicated. (B) Western blot of 293T cells transduced with vectors expressing Flag-SUMO2 or Flag-SUMO2ΔGG. SUMO1ΔGG expression is shown for comparison.

To determine if SUMO2 is also poorly expressed in embryonic cells, we inserted Flag-tagged SUMO2 and SUMO2ΔGG into the same pLVX lentiviral vector as was used for the SUMO1 constructs. F9, E14, and 293T cells were transduced with the SUMO2 or SUMO2ΔGG vectors and selected for drug resistance. Cell lysates were prepared and examined for SUMO2 or SUMO2ΔGG expression by Western blotting. F9 cells expressed free SUMO2 and SUMO2ΔGG proteins to roughly similar high levels, while free SUMO1 protein levels were dramatically lower than SUMO1ΔGG expression levels, as before (Fig. 3A, left blot). Embryonic E14 cells also expressed free SUMO2 and SUMO2ΔGG to similar levels, in contrast to the very low expression of free SUMO1 relative to SUMO1ΔGG (Fig. 3A, right blot). Differentiated 293T cells transduced with the SUMO2 and SUMO2ΔGG vectors expressed the two constructs to similar levels, as expected (Fig. 3B). These results indicate that poor expression of free SUMO in embryonic cells occurs specifically for SUMO1 and not for all the SUMO family members.

### Reducing SUMO1 conjugation activity restores free SUMO1 expression.

**FIG 4:**
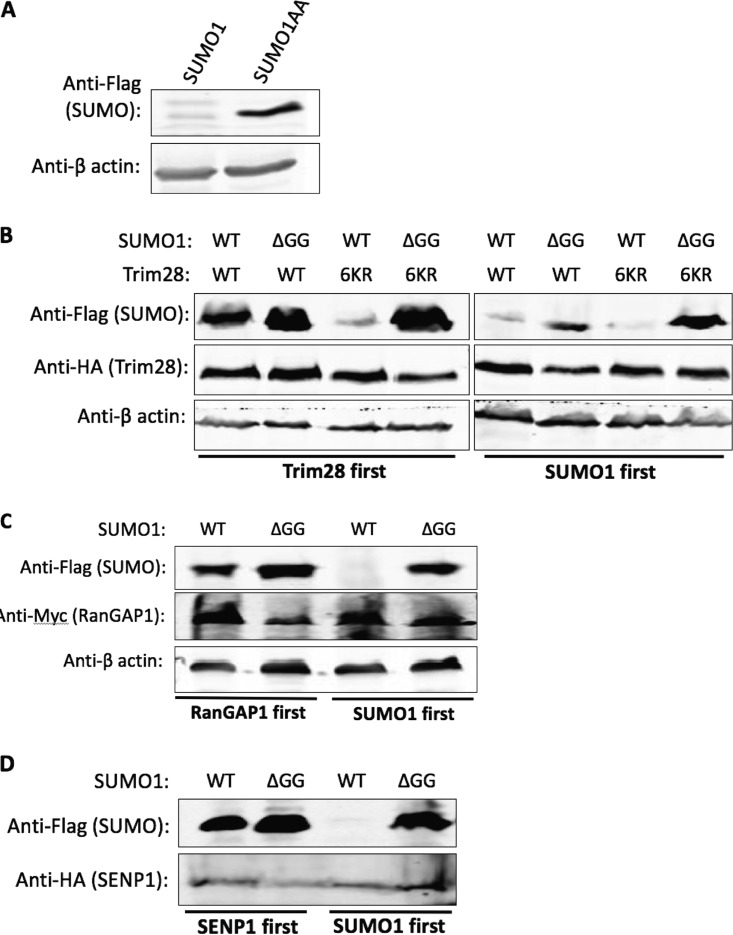
Free SUMO1 accumulation is allowed if conjugation to endogenous substrates is prevented or limited. (A) Western blot of F9 cells transduced with vectors expressing Flag-SUMO1 or Flag-SUMO1AA. (B) Western blot analysis of F9 cells engineered by successive transductions to coexpress Flag-SUMO1 (WT) or Flag-SUMO1ΔGG (ΔGG) along with HA-Trim28 (WT) or HA-Trim28^6KR^ (6KR), as indicated. Blots were probed with anti-Flag antibodies to detect SUMO1 and anti-HA antibodies to detect Trim28. The right four lanes show cells transduced with SUMO1 first and Trim28 second, and the left four lanes show cells transduced with Trim28 first and SUMO1 second. Prior expression of Trim28 allowed good expression of WT SUMO1; subsequent expression was not effective. (C) Western blot analysis of F9 cells engineered by successive transductions to coexpress Flag-SUMO1 (WT) or Flag-SUMO1ΔGG (GG) along with Myc-RanGAP1. Blots were probed with anti-Flag antibodies to detect SUMO1 and with anti-Myc antibodies to detect RanGAP1. The right two lanes show cells transduced with SUMO1 first, and the left two lanes show cells transduced with RanGAP1 first. Prior expression of RanGAP1 allowed good expression of WT SUMO1; subsequent expression was not effective. (D) Western blot analysis of F9 cells coexpressing Flag-SUMO1 and HA-SENP1. The blot was probed with anti-Flag antibodies to detect SUMO1, and with anti-HA antibodies to detect SENP1. The left two lanes show cells transduced with SENP1 first, and the right two lanes show cells transduced with SUMO1 first.

SUMO1ΔGG is missing the last six amino acids of SUMO1, including the diglycine residues necessary for conjugation to substrates. To test if this C-terminal tail was a target for posttranscriptional regulation, we created a construct expressing full-length Flag-tagged SUMO1 but containing alanine substitutions of the diglycine residues (SUMO1AA) that would prevent conjugation. F9 cells were transduced with the SUMO1 or SUMO1AA vector, and lysates were prepared and examined for SUMO expression by Western blotting. Free SUMO1AA was expressed well in embryonic cells to levels similar to those seen with SUMO1ΔGG and in contrast to the poor accumulation of free SUMO1 (Fig. 4A). This result suggests that the toxic effects of SUMO1 overexpression required SUMO1 conjugation to at least some substrates.

If elevated levels of particular SUMO1-conjugated substrates are toxic to F9 cells, then reducing levels of conjugation to those substrates might allow free SUMO1 to be more highly expressed. In one approach designed to reduce SUMO1 conjugation of endogenous substrates, we coexpressed SUMO1 with a highly sumoylated protein that could act as a competitive “sponge” for the overexpressed SUMO1. Tripartite motif-containing 28 (Trim28 or KAP1), a transcriptional repressor protein, is one such highly sumoylated protein, with six lysine residues that can be conjugated by SUMO (20). Hemagglutinin (HA)-tagged Trim28 cDNA was inserted into the same pLVX lentiviral vector used for the SUMO constructs, but encoding G418 resistance, with the *neoR* gene in place of the *puroR* gene. We also designed a construct expressing Trim28 in which all six lysine residues known to act as SUMO acceptors were mutated to arginine, rendering the mutant unavailable for SUMO conjugation (Trim28^6KR^). SUMO and Trim28 constructs was introduced into cells sequentially. F9 cells were transduced and selected for the expression of the Trim28 constructs, followed by transduction and selection for the expression of the SUMO constructs, or in the reverse order. Cell lysates were prepared from these doubly drug-resistant cells, and SUMO1 expression levels were assessed by Western blotting.

Cells that were first transduced and selected for expression of the Trim28 constructs before subsequent transduction with the SUMO vectors were profoundly different with respect to the ability to express free SUMO1. Overexpression of wild-type Trim28 (Trim28_WT_), but not Trim28_6KR_, allowed greatly increased subsequent detection of free SUMO1 (Fig. 4B, lanes 1 to 4). Thus, the accumulation of free SUMO1 required that the overexpressed Trim28 be capable of serving as a SUMO1 acceptor, consistent with its serving as a SUMO1 “sponge.” In contrast, cells that were first transduced and selected for the expression of SUMO1 before subsequent transduction with the Trim28 vectors continued to show weak expression of free SUMO1 even in the presence of exogenous Trim28_WT_ or Trim28_6KR_ proteins (Fig. 4B, lanes 5 to 8). Thus, later expression of Trim28 could not reverse the mechanism that prevents accumulation of free SUMO1. As expected, SUMO1ΔGG was well expressed with or without the Trim28 proteins.

Trim28 can act as an E3 ligase in the SUMO conjugation cascade (21), mediates autosumoylation (20), and contains important functions for embryonic cells (22, 23). Although we found no evidence to suggest that Trim28 overexpression had any adverse effects on embryonic cells, the rescue of SUMO1 expression might have involved some specific Trim28 function. To evaluate this possibility, we repeated the previous experiment using a different candidate SUMO “sponge.” Ran GTPase-activating protein 1 (RanGAP1) is a regulatory trafficking protein that is commonly used in SUMO1 studies and was one of the earliest proteins to have been found to be a target of SUMO1 conjugation (5). Myc-tagged RanGAP1 cDNA was inserted into a pLVX lentiviral vector containing the G418 resistance drug selection marker. As with the Trim28 constructs, F9 cells were transduced and selected for the expression of the RanGAP1 construct before or after transduction by the SUMO1 or SUMO1ΔGG constructs. Lysates were prepared and SUMO1 expression levels were examined by Western blotting probing for the Flag tag. Expression of free SUMO1 improved markedly when RanGAP1 was overexpressed prior to SUMO1 overexpression but not when RanGAP1 was expressed after SUMO1 (Fig. 4C). Thus, the block to accumulation of free SUMO1 can be relieved by prior overexpression of multiple SUMO substrates.

In another approach designed to reduce the levels of SUMO1 conjugation to substrates, we tested the overexpression of SUMO-specific peptidase 1 (SENP1). HA-tagged SENP1 was cloned into a pLVX vector, and F9 cells were transduced and selected for the expression of SENP1 either before or after transduction and selection for the expression of the SUMO constructs. Lysates were prepared and analyzed for SUMO1 expression by Western blotting. SENP1 overexpression prior to expression of SUMO1 dramatically improved levels of free SUMO1 accumulation, almost reaching SUMO1ΔGG expression levels (Fig. 4D). However, SUMO1 overexpression levels did not improve when this order was reversed.

In sum, we show here that embryonic cells do not tolerate overexpression of SUMO1 and that cells surviving transduction specifically downregulate the accumulation of free SUMO1 at a posttranscriptional stage, maintaining SUMO1 in the form of selected high-molecular-weight conjugates. Reducing SUMO1 conjugation by mutation of the diglycine residues or by overexpression of a SUMO sponge or by overexpression of SENP1 dramatically increased the expression of free SUMO1. The results suggest that the toxic effects of SUMO1 overexpression are a consequence of the accumulation of SUMO1-modified proteins and not of the accumulation of free SUMO1 protein itself. Moreover, the embryonic cells surviving the forced overexpression become committed to the altered distribution of SUMO1 irreversibly, since reducing the levels of SUMO1-modified proteins after the pattern of distribution had been established did not rescue the accumulation of free SUMO1.

## DISCUSSION

In this report, we have provided evidence that embryonic cells do not readily tolerate forced overexpression of SUMO1 likely occurring through the effects of inappropriately high levels of SUMO1-modified substrates. Embryonic cells surviving after transduction by expression constructs have an aberrant distribution of SUMO1 between free and conjugated pools and do not accumulate free conjugation-competent SUMO1 (Fig. 1). In contrast, conjugation-defective SUMO1 mutants could be readily expressed as free protein. The redistribution of SUMO was specifically observed with SUMO1 and not SUMO2. We do not yet know the mechanism of action responsible for the altered processing of SUMO1 in cells surviving transduction, but the analysis of SUMO1 DNA and mRNA levels suggests that the changes are posttranscriptional. The relatively high frequency of recovery of surviving cells after transduction (about 10% of control) is not consistent with mutation in the host genome but rather suggests an adaptation through altered physiology or processing of SUMO1. We cannot completely rule out the possibility that there has been selection for subtle mutations in the SUMO1 expression construct that are responsible for the altered distribution, but no common mutations were detected in DNA sequences of pooled transgenes.

**FIG 5:**
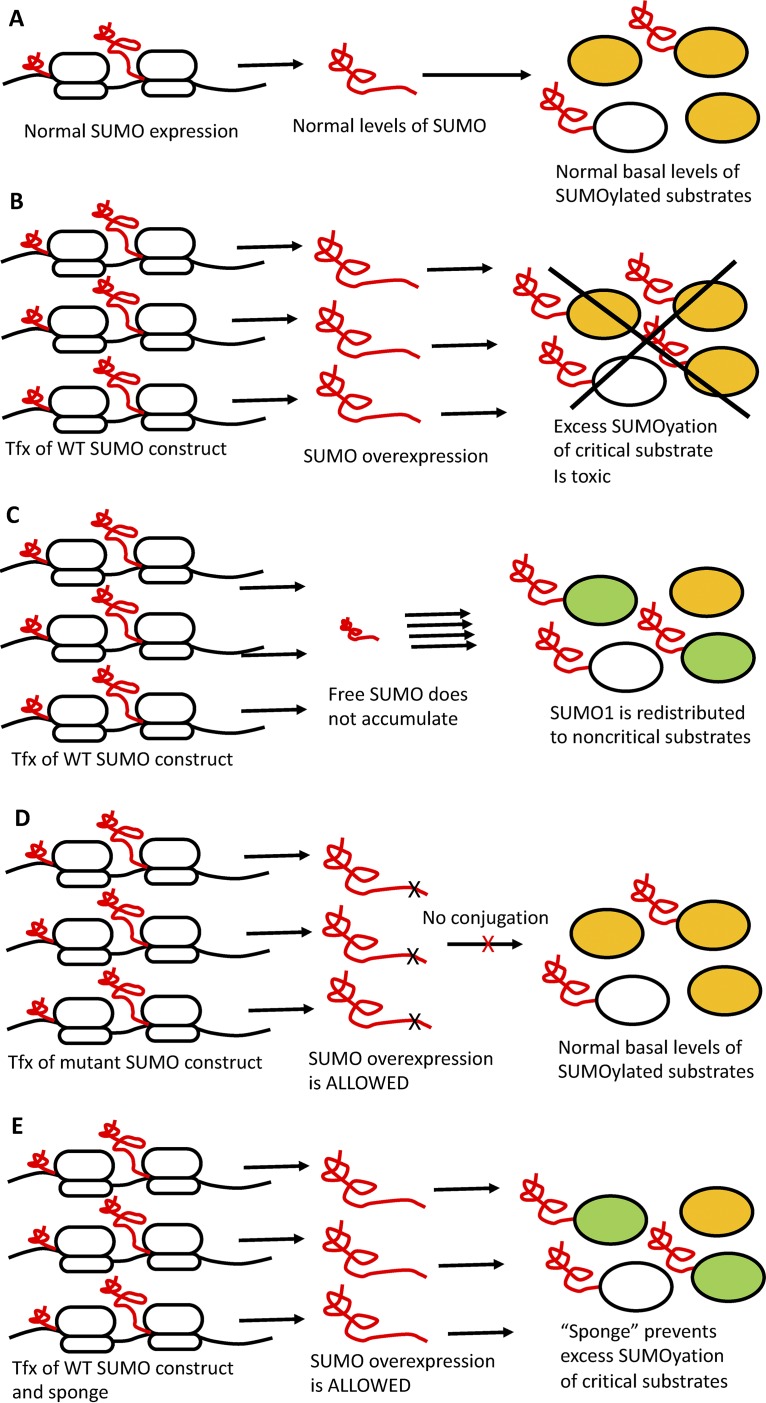
Model for SUMO1 expression and conjugation to substrates in embryonic cells after transfection with various expression constructs. (A) Normal expression of SUMO1 (shown being translated, in red) results in normal levels of free SUMO1 and normal levels of sumoylated substrates (white for irrelevant and orange for critical substrates). (B) Forced overexpression of WT SUMO1 and formation of oversumoylated critical substrates (orange) is toxic and is not tolerated. Tfx, transfection. (C) Cells selected for retention of SUMO1 expression construct exhibit redistribution of SUMO1, with no accumulation of free SUMO1 and altered substrate utilization, limiting levels of sumolyated critical substrates. (D) Overexpression of SUMO1 mutants that cannot be conjugated does not result in accumulation of oversumoylated critical substrates and is not toxic, and accumulation of free SUMO1 is allowed. (E) Overexpression of WT SUMO1 in the presence of a SUMO “sponge” prevents excess sumoylation of critical substrates and so is again allowed.

In principle, elevated levels of either free SUMO1 or SUMO1-modified proteins could be problematic with respect to high expression in embryonic cells. We found that SUMO1 mutants that could not conjugate to substrates (SUMO1ΔGG and SUMO1AA) were readily expressed in embryonic cells to high levels (Fig. 1A and B and 4A), suggesting that the accumulation of free SUMO1 protein *per se* was not toxic but rather was tolerated so long as the free SUMO1 protein could not be conjugated to substrates. To test whether SUMO1 conjugated substrates initiated the altered utilization, we blocked or reduced SUMO1 conjugation to endogenous substrates using various approaches. One approach was to coexpress SUMO1 with a highly sumoylated protein to act as a “SUMO sponge” for excess SUMO1 protein. We chose RanGAP1 and Trim28 as SUMO sponges because Trim28 is known to contain multiple sites for sumoylation (20), and RanGAP1 was one of the first known prominent substrates of sumoylation (24). In the background of Trim28 and RanGAP1 overexpression, expression of free SUMO1 in embryonic cells was greatly improved (Fig. 4B and data not shown). Another approach was to overexpress SUMO1 along with SENP1, the enzyme responsible for deconjugation of SUMO1 from its substrates (16). We found that overexpression of SENP1 greatly improved exogenous SUMO1 expression as well. These results indicate that SUMO1 overexpression in embryonic cells is prevented as a response to the accumulation of SUMO1-modified proteins and not as a response to the overexpression of SUMO1 protein *per se* (see Fig. 5 for model).

An intriguing aspect of these experiments is that free SUMO1 accumulation improved only when the SUMO sponge or SENP1 was overexpressed prior to transduction with the SUMO1 vector. Reversing this order did not result in improved accumulation of free SUMO1; once the pattern of utilization was established, it was not relieved by subsequent lowering of SUMO1 conjugation. SUMO1 modification of substrates was previously reported to have had long-term effects even when SUMO1 was no longer conjugated to the substrate (24), and perhaps the SUMO1 modification of certain critical embryonic substrates creates long-term effects that are not immediately reversible. Though none were detected, any mutations of the SUMO1 transgene selected for during transduction would also not be reversed.

The results described here suggest that the accumulation of SUMO1-modified proteins was toxic or inhibited replication or caused cell death and that only a few cells survived transduction by expressing low levels of free SUMO1. One possible explanation for the observations would be the sequestration of the SUMO1 in an intracellular location that precludes its extraction, but we consider this unlikely given the harsh conditions used for lysis. We favor the possibility that an increase in the levels of specific SUMO1-modified proteins triggers a mechanism for lowering free SUMO1 expression in the surviving embryonic cells. This mechanism could in principle act at any of several stages of expression: at the retention of the transgene, at transcription, at translation, or at a posttranslational step. Examination of the SUMO1 transgene and transcript levels in embryonic cells transduced with the SUMO1 vector showed that both DNAs and RNAs were present at levels equivalent to those in embryonic cells transduced with the SUMO1ΔGG vector (Fig. 2A and C). Thus, the likely mechanisms are posttranscriptional and could have involved protein stability. We found no evidence for SUMO1 protein degradation by the proteosomal or lysosomal pathways (Fig. 2D). The most likely mechanism is an altered course of SUMO1 processing, with all the detectable SUMO1 being distributed to high-molecular-weight conjugates that are tolerated, and with no accumulation of free SUMO1. This could be achieved by redirecting the SUMO1 to acceptable substrates or by increasing the levels of these substrates or by decreasing the levels of critical substrates that become toxic to cell viability upon excessive SUMO1 conjugation.

A striking aspect of the SUMO redistribution is the specificity for SUMO1 and not another SUMO family member. SUMO2 was overexpressed in both embryonic and differentiated cells as efficiently as SUMO1ΔGG and accumulated to high levels (Fig. 2A). It is possible that SUMO1 is redistributed because it is more actively conjugated to critical substrates whereas SUMO2 is less efficiently conjugated or because addition of SUMO1 to critical substrates is inherently more toxic. The results are consistent with previous studies showing that SUMO1 knockout is tolerated (13) but that the increase in steady-state levels of SUMO1 resulting from SENP1 knockout is embryonic lethal (16). The fact that the cells adapt to SUMO1 overexpression by reducing SUMO1 availability for conjugation suggests that an alternative mechanism of escape—reducing the levels of the critical substrate itself—is not a viable option. The identity of the crucial substrate (or substrates) in embryonic cells is not known, but several proteins known to be modified by addition of SUMO1 are candidates. These include regulators of stem cell differentiation such as Oct4 and Sox2 (25), which control levels of expression of the Nanog protein, and it is plausible that inappropriate levels of their modification could be toxic. Further studies exploring the distribution of SUMO1 to its substrates in embryonic cells will be important for understanding the complex nature of embryonic cells.

## MATERIALS AND METHODS

### Cell lines.

F9, PCC4, and NIH 3T3 cells were cultured in Dulbecco’s modified Eagle medium (DMEM) with 10% fetal bovine serum (FBS), 100 U/ml penicillin, 0.05 mM streptomycin, and 2 mM l-glutamine. E14 cells were cultured in DMEM with 15% FBS, 20 mM HEPES, 0.1 mM nonessential amino acids, 0.1 mM 2-mercaptoethanol, 100 U/ml penicillin, 0.05 mM streptomycin, 2 mM l-glutamine, and leukemia inhibitory factor added fresh at the time of culture. Plates were coated with 0.1% gelatin prior to plating of ES or EC cells. Retinoic acid (RA) (Sigma) (1 μM) was used for induced differentiation of EC cells.

### Plasmid construction.

SUMO1, SUMO2, and mutant cDNAs were cloned into the pLVX-EF1a-IRES vector carrying the puromycin resistance gene (*puroR*) (Clontech). Trim28 and RanGAP1 cDNAs were inserted into versions of the pLVX-EF1a-IRES vector expressing G418 resistance in which the *puroR* gene has been replaced with the *neoR* gene. pVSV-G was obtained from Addgene (pMD2.G). pCMVR8.2 was a gift from Didier Trono (Addgene plasmid no. 12263).

### Viral preparation and transduction.

Viruses were produced in 293T cells. A total of 3.5 × 10^6^ 293T cells were plated in 10-cm-diameter dishes. The following day, 293T cells were transfected with 8 μg of a particular pLVX vector, 4 μg of pCMVR8.2 DNA, and 4 μg of pVSV-G DNA using polyethylenimine (PEI). Culture supernatants were collected for virus preparations as previously described (26).

### Cell colony formation assay.

A total of 10^5^ F9 cells were plated in 6-well plates. The following day, cells were infected with virus containing 8 μg/ml Polybrene for 3 h. Virus was washed off and replaced with fresh medium. At 48 h later, medium with drug selection was added to cells and uninfected cells were left to die over ∼2 weeks. When colonies were visible, cells were washed with Dulbecco’s phosphate-buffered saline (DPBS) and incubated with 100% methanol at −20°C for 10 min. Methanol was washed off, and cells were dyed with Giemsa staining.

### Lysate preparation.

Cells were collected and washed in ice-cold DPBS. Cells were lysed either with harsher radioimmunoprecipitation assay (RIPA) buffer (1% NP-40, 1% sodium deoxycholate, 0.1% SDS, 150 mM NaCl, 25 mM Tris-HCl, pH 7.6) where specified or with milder 0.1% NP-40 lysis buffer (0.1% NP-40, 250 mM NaCl, 20 mM sodium phosphate [pH 7.0], 30 mM sodium pyrophosphate, 5 mM EDTA, 10 mM NaF), with both containing 1× complete protease inhibitor (Roche). Lysis buffer was added to the pellet at twice the cell pellet volume. Cells were lysed on ice for 30 min, and the lysates were clarified by centrifugation at 14,000 rpm for 15 min at 4°C. Lysates were used fresh or stored at −80°C.

### PCR.

PCRs were conducted using KOD Hot Start DNA polymerase (EMD Millipore) according to manufacturer’s instructions. For qRT-PCRs, RNA was extracted from cells using an RNeasy minkit (Qiagen) according to the manufacturer’s instructions. cDNA was prepared using a High-Capacity cDNA reverse transcription kit (Applied Biosystems) according to the manufacturer’s instructions. For qPCRs, total DNA was isolated using a DNeasy kit (Qiagen) according to the manufacturer’s instructions. DNA isolates or cDNAs were combined with FastStart universal SYBR green master mix (Roche) containing 300 nM concentrations of the indicated primers. qPCR was performed in 96-well plates using LightCycler 96 (Roche) under the following reaction conditions: 10 min at 95°C, followed by 45 cycles of 30 s at 95°C, 30 s at 60°C, and 30 s at 72°C.

The primer sequences used for PCR, qPCR, and qRT-PCR were as follows. The PCR primers for amplifying transgene insertions in the pLVX-EF1 vector were pLVX-F (TCAAGCCTCAGACAGTGGTTC) and pLVX-R (ACCCCTAGGAATGCTCGTCAAGAA). The qPCR primers for DNA levels were SUMO1-F (ATTGGACAGGATAGCAGTGAGA) and SUMO1-R (TCCCAGTTCTTTCGGAGTATGA), hGAPDH F (ACATCATCCCTGCCTCTAC) and hGAPDH R (TCAAAGGTGGAGGAGTGG), mCyclophilin A-F (GCAGGTCCATCTACGGAGAGAAA) and mCyclophilin A-R (GTCAACAGATCCCATTCACTGTTTCTTA), and Puro-F (GCCGCGCAGCAACAGAT) and Puro-R (CGCTCGTAGAAGGGGAGGTT). The qRT-PCR primers for RNA levels were mSUMO1-F (ATTGGACAGGATAGCAGTGAGA) and mSUMO1-R (TCCCAGTTCTTTCGGAGTATGA), mSUMO2-F (TGGAGTAAAGTAGCAGGCTCCCTTT) and mSUMO2-R (ACTAATGAAAGCCTATTGTGAAC), and mGAPDH-F (AACGACCCCTTCATTGAC) and mGAPDH-R (TCCACGACATACTCAGCAC).

### Antibodies.

Western blotting used the following reagents: anti-SUMO antibody (sc-9060, Santa Cruz Biotechnology), anti-HA.11 (901515, BioLegend), anti-myc 71D10 (2278, Cell Signaling Technology), anti-myc 9E10 (sc-40, Santa Cruz Biotechnology), anti-Flag M2 (F3165, Sigma-Aldrich), and anti-β-actin (A1978, Sigma).
